# IL-21–producing effector Tfh cells promote B cell alloimmunity in lymph nodes and kidney allografts

**DOI:** 10.1172/jci.insight.169793

**Published:** 2023-10-23

**Authors:** Hengcheng Zhang, Cecilia B. Cavazzoni, Manuel A. Podestà, Elsa D. Bechu, Garyfallia Ralli, Pragya Chandrakar, Jeong-Mi Lee, Ismail Sayin, Stefan G. Tullius, Reza Abdi, Anita S. Chong, Bruce R. Blazar, Peter T. Sage

**Affiliations:** 1 Transplantation Research Center, Renal Division, Brigham and Women’s Hospital, Harvard Medical School, Boston, Massachusetts, USA.; 2 Department of Surgery, Section of Transplantation, University of Chicago, Chicago, Illinois, USA.; 3 Division of Transplant Surgery & Transplant Surgery Research Laboratory, Department of Surgery, Brigham and Women’s Hospital, Harvard Medical School, Boston, Massachusetts, USA.; 4 Department of Pediatrics, Division of Blood & Marrow Transplant & Cellular Therapies, University of Minnesota, Minneapolis, Minnesota, USA.

**Keywords:** Immunology, Transplantation, Adaptive immunity, Immunoglobulins, T cells

## Abstract

Follicular helper T (Tfh) cells have been implicated in controlling rejection after allogeneic kidney transplantation, but the precise subsets, origins, and functions of Tfh cells in this process have not been fully characterized. Here we show that a subset of effector Tfh cells marked by previous IL-21 production is potently induced during allogeneic kidney transplantation and is inhibited by immunosuppressive agents. Single-cell RNA-Seq revealed that these lymph node (LN) effector Tfh cells have transcriptional and clonal overlap with IL-21–producing kidney-infiltrating Tfh cells, implicating common origins and developmental trajectories. To investigate the precise functions of IL-21–producing effector Tfh cells in LNs and allografts, we used a mouse model to selectively eliminate these cells and assessed allogeneic B cell clonal dynamics using a single B cell culture system. We found that IL-21–producing effector Tfh cells were essential for transplant rejection by regulating donor-specific germinal center B cell clonal dynamics both systemically in the draining LN and locally within kidney grafts. Thus, IL-21–producing effector Tfh cells have multifaceted roles in Ab-mediated rejection after kidney transplantation by promoting B cell alloimmunity.

## Introduction

Kidney transplantation is the most frequent type of transplant procedure, accounting for approximately 80% of all solid organ transplant surgeries (1). However, Ab-mediated rejection (ABMR) restricts the long-term survival of allografts after kidney transplantation, despite recent developments in broad immunosuppression (2, 3). In ABMR, donor-specific Abs (DSAs) bind to donor graft antigens leading to endothelial damage, intimal arteritis, and graft loss (4). A fundamental paradigm in the treatment of ABMR is to attenuate DSAs, but strategies to do so have not been fully effective (5, 6). Ab responses are tightly controlled during the germinal center (GC) reaction, where T follicular helper (Tfh) cells stimulate B cells to undergo class switch recombination (CSR) and somatic hypermutation (SHM), thereby enhancing Ab functions (7, 8). Recent evidence suggests a direct relationship between Tfh cells and DSAs as well as rejection in patients (9–11). In addition, a recent report suggests that eliminating total Tfh cells after allogeneic kidney transplantation in preclinical models results in prolonged survival (12).

Although Tfh cells have been implicated in controlling rejection after transplantation, the origins and precise functions of these cells in allogeneic B cell responses and rejection pathology are relatively unknown. A current paradigm suggests that Tfh cells are a uniform population; however, newer data suggest that Tfh cells can exist as unique subsets with distinct roles in disease progression (13). In some cases, these Tfh subsets have been denoted by their cytokine production. For instance, a subset of circulating Tfh cell (ICOS^+^CXCR3^+^, Tfh1 cells) correlates with high avidity Abs after influenza infection/vaccination (14, 15). In patients with SARS-CoV-2, Tfh1 cells positively and Tfh17 cells negatively correlate with Ab responses (16). We and others have found that Tfh13 cells have specialized roles in promoting IgE and allergic airway disease (17, 18). The cytokine IL-21 has important roles in mediating B cell responses, but only a fraction of Tfh cells produce IL-21 (19, 20). Although Tfh cells are thought to predominantly control B cell responses through interactions in lymphoid organs, Tfh-like cells can also be found in human kidney allografts (21). Importantly, clonally expanded pathogenic B cells can also be found in kidney and heart allografts, and their lack of clonal overlap with draining lymph nodes (dLNs) and circulating B cells suggests a distinct local contribution to ABMR (22, 23).

Here, we investigated the origins and functions of effector Tfh cells in controlling ABMR in kidney allografts. Using an IL-21 fate-mapping mouse, we found potent differentiation of IL-21–producing effector Tfh cells in LNs and kidney allografts. Using single-cell RNA-Seq (scRNA-Seq) with matched T cell receptor (TCR) profiling, we found a substantial transcriptional and clonal overlap of IL-21–producing effector Tfh cells from dLNs with graft-infiltrating IL-21–producing Tfh-like cells, suggesting similar origins and parallel developmental trajectories. We used a deleter mouse for IL-21–producing Tfh cells along with a single-cell B cell assay to assess the systemic and graft-specific roles of IL-21–producing Tfh cells in alloreactive B cell GC clonal dynamics. We found that selective deletion of these cells in vivo resulted in prolonged survival, reduced pathological allograft damage, reduced DSA-specific B cell clonal dynamics in LNs and grafts, and reduced B cell SHM in LNs only. Together, these data reveal that a specific subset of IL-21–producing effector Tfh cell controls B cell alloimmunity both systemically and locally within kidney allografts.

## Results

### IL-21–producing Tfh cells are potently generated after allogeneic kidney transplantation.

**Figure 1:**
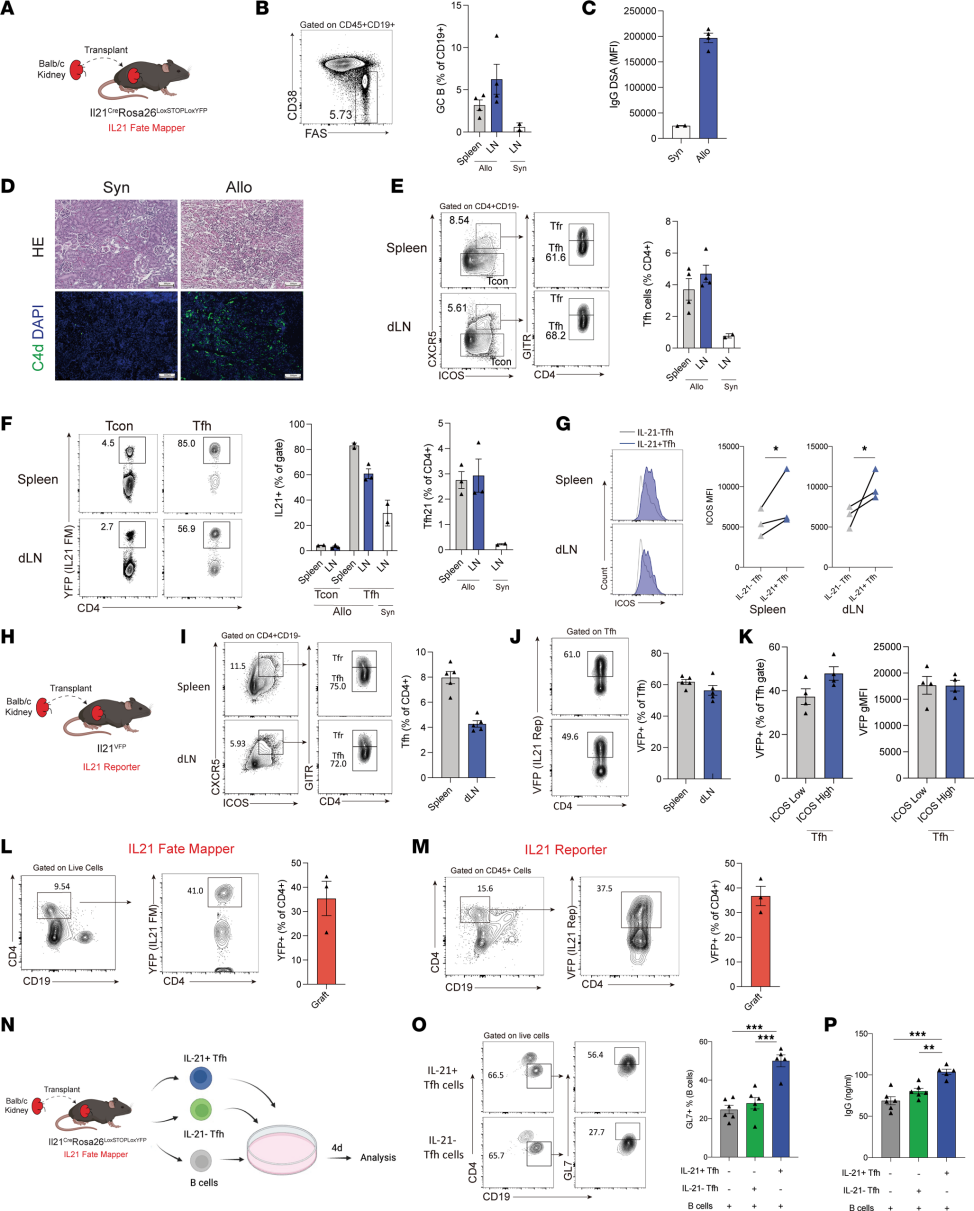
IL-21–producing Tfh cells are generated in LNs and grafts after allogeneic kidney transplantation. (**A**) Schematic of kidney transplantation. (**B**) Gating strategy (left) and quantification of FAS^+^CD38^–^ GC B cells (right) in the spleen and dLN. (**C**) DSA IgG measurements from serum. (**D**) Histology and C4d IF in grafts. Scale bars: 100 μm; original magnification: ×100. (**E**) Quantification of Tfh (CD4^+^CD19^–^CXCR5^+^ICOS^+^GITR^–^) cells in the spleen and dLN. Representative gating (left) and quantification (right) are shown. (**F**) Gating strategy (left) and quantification (right) of IL-21 fate mapped (YFP^+^) Tcon and Tfh cells. Gated on CD4^+^CD19^–^CXCR5^+^ICOS^+^ cells. (**G**) Expression of ICOS in IL-21 fate-mapped (IL-21^+^) or nonfate-mapped (IL-21^–^) Tfh cells. (**H**) Schematic of kidney transplantation of Balb/c kidneys into IL-21 direct reporter mice. (**I**) Gating strategy (left) and quantification (right) of total Tfh cells. (**J**) Gating strategy (left) and quantification (right) of IL-21–expressing (VFP^+^) Tfh cells. (**K**) Frequency (left) and MFI (right) of VFP^+^ cells in ICOS low and high Tfh cells. gMFI, geometric MFI. (**L** and **M**) Quantification of IL-21–producing (YFP^+^, and VFP^+^) graft-infiltrating CD4^+^ T cells. In **A**–**M**, data are combined from 2 independent experiments, *n* = 2–5 mice replicates per group. (**N**) Schematic of in vitro Tfh-mediated B cell stimulation assay. IL-21 fate-mapped (IL-21^+^), nonfate-mapped (IL-21^–^) Tfh, and B cells from recipients were cocultured for 4 days with anti-CD3/IgM. (**O**) Gating strategy (left) and quantification (right) of activated (GL7^+^) B cells. (**P**) IgG concentration in culture supernatants of the in vitro assays. In **O** and **P**, data are from 1 experiment and are representative of 2 independent experiments, *n* = 5–6 wells per group. Student’s 2-tailed unpaired *t* test for **G**, 1-way ANOVA with Tukey’s multiple comparison test for **O** and **P**. *P < 0.05; **P < 0.01; ***P < 0.001.

Despite the association of Tfh cells with kidney transplant rejection in both patients and in preclinical models, little is known about the origins and precise functions of Tfh cell subsets in mediating disease progression. Part of this is due to a lack of preclinical models to investigate Tfh subsets and their responses in both LN and kidney allograft effector sites. Therefore, we used tools to identify and track Tfh cells that have previously produced IL-21 by using *Il21*^Cre^ *Rosa26*^Lox-STOP-Lox-YFP^ fate-mapping mice that mark cells that have expressed IL-21 previously with yellow fluorescent protein (YFP) expression (24) We transplanted a kidney from Balb/c (allogeneic) or C57BL/6 (B6) (syngeneic) mice into *Il21*^Cre^*Rosa26*^Lox-STOP-Lox-YFP^ recipients using a previously published allogeneic kidney transplant procedure (12, 22). The spleen, dLN, and kidney graft were harvested for analysis at day 20 following transplantation, which corresponds to a time point just prior to full rejection (Figure 1A). GC B cells were potently induced in both the spleen and the dLN of allograft recipients (Figure 1B) and was associated with higher IgG DSA levels and ABMR damage consisting of tubulitis, glomerulitis, and C4d deposition (a pathological marker of ABMR) in kidney allografts (Figure 1, C and D). The frequency of total CD4^+^CXCR5^+^GITR^–^ Tfh cells was also increased in mice transplanted with an allogeneic kidney in dLN (Figure 1E). To identify Tfh cells that have ever produced IL-21, we gated on YFP^+^ cells within the Tfh population. Approximately 60% of Tfh cells from the allogeneic dLN (and approximately 80% from the spleen) had evidence of prior IL-21 production, which was in contrast to approximately 20% in Tfh from syngeneic dLNs and only 3% in CD4^+^CXCR5^–^ T conventional (Tcon) cells from the allogeneic dLN (Figure 1F). In addition, IL-21 fate-mapped Tfh cells displayed higher expression levels of ICOS compared with nonfate-mapped Tfh subsets in both spleen and dLN, suggesting a distinct phenotype (Figure 1G).

To assess the frequency of Tfh cells that are *currently* producing IL-21, we performed similar kidney transplantation experiments in an *Il21*^VFP^ direct reporter mouse strain (Figure 1H). Consistent with the results from the fate-mapping system, IL-21–producing Tfh cells comprised approximately 50% of all Tfh cells in the dLN and approximately 60% within the spleen (Figure 1, I and J). The current IL-21 expression in Tfh cells was not indicative of a heightened activation state since Tfh cells subdivided into ICOS^hi^ or ICOS^lo^ populations had similar percentages and total levels of IL-21 (Figure 1K). Since Tfh-like cells have been reported to be present within kidney allografts in humans, we further assessed IL-21 responses in graft-infiltrating CD4^+^ T cells. We found approximately 35% of the CD4^+^ T cells in kidney grafts produce IL-21 since they are positive for the fate-mapper (YFP) or direct reporter (vivid verde fluorescent protein [VFP]) fluorophores (Figure 1, L and M).

We conducted an in vitro functional assay to explore the functional difference of LN IL-21 fate-mapped Tfh compared with nonfate-mapped Tfh cells (Figure 1, N–P). Tfh cells that had produced IL-21 previously were sorted (as YFP^+^ Tfh cells) from dLNs of allogeneic transplantation recipients and cultured with B cells and anti-CD3/IgM. IL-21 fate-mapped Tfh cells stimulated approximately 50% of B cells to express the B cell activation antigen GL7, which was in contrast to approximately 28% by nonfate-mapped Tfh cells. Moreover, IL-21 fate-mapped Tfh cells stimulated more B cell IgG secretion compared with nonfate-mapped Tfh cells, suggesting increased ability to drive humoral immunity. IL-21 fate-mapped Tfh cells also showed evidence of alloreactivity (Supplemental Figure 1; supplemental material available online with this article; https://doi.org/10.1172/jci.insight.169793DS1). Together, these data demonstrate that after allogeneic kidney transplantation, a distinct subset of Tfh cells marked by IL-21 expression emerges in the dLN and spleen, as well as in the kidney allograft, that can potently induce Ab secretion.

### Overlapping origins and parallel developmental trajectories of LN and kidney allograft Tfh cells.

**Figure 2:**
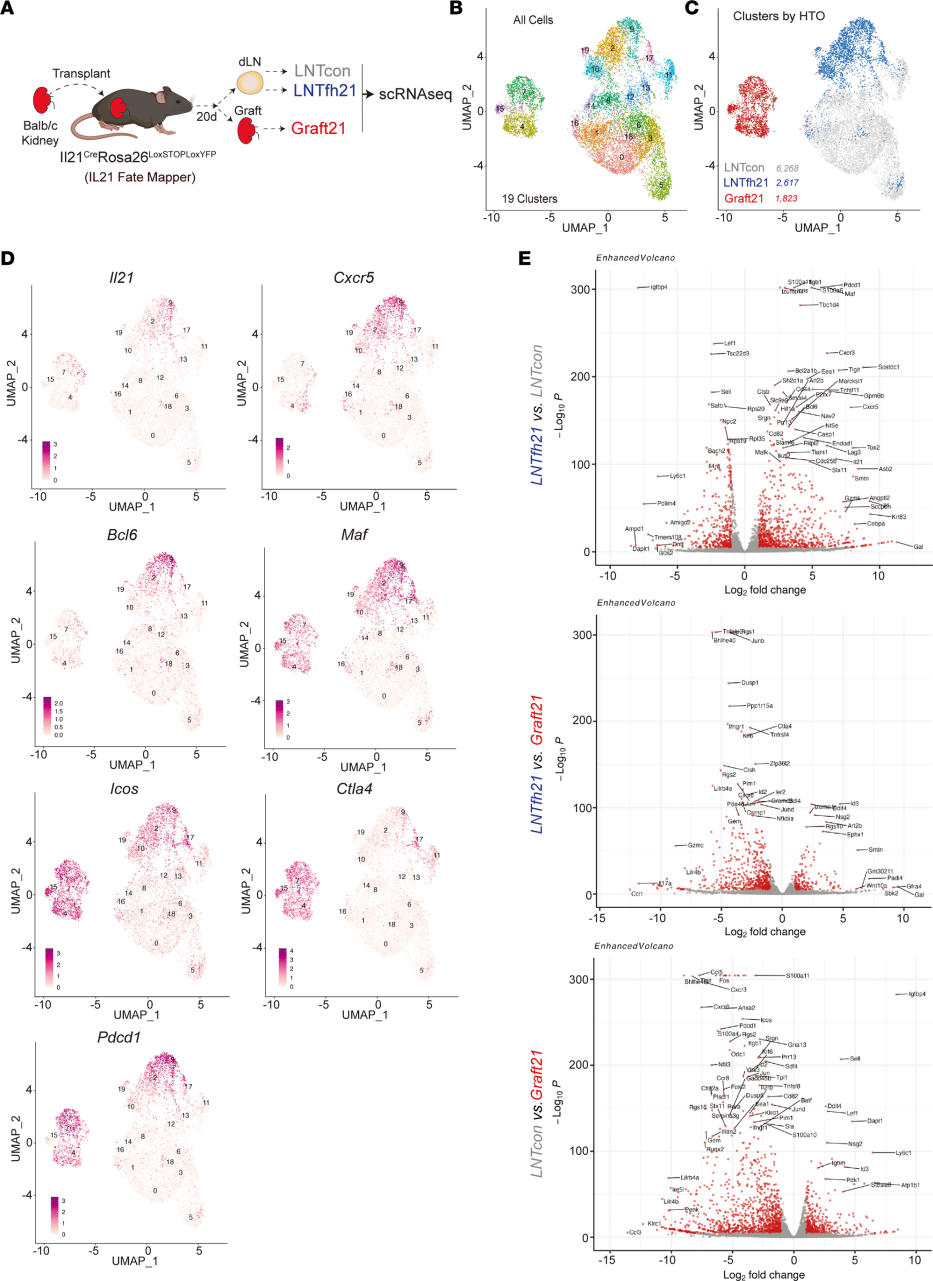
Transcriptional features of LN and graft IL-21–producing Tfh cells. (**A**) Diagram of the scRNA-Seq experiment. Balb/c kidneys were transplanted into *Il21*^Cre^*Rosa26*^Lox-STOP-Lox-YFP^ (IL-21 fate mapping) mice. Twenty days after transplantation, Tcon (CD4^+^CXCR5^–^YFP^–^, LNTcon), Tfh21 (CD4^+^CXCR5^+^YFP^+^, LNTfh21) from dLN and graft-infiltrating IL-21–producing cells (CD4^+^YFP^+^, Graft21) were sorted and scRNA-Seq was performed. (**B**) UMAP plot showing unsupervised clustering of all postfilter cells. (**C**) Cells from UMAP marked by group by HTO expression. Total number of cells per group is indicated. (**D**) Feature plots showing indicated gene expression levels. (**E**) Volcano plots showing DEGs between indicated groups.

The shared expression of IL-21 by dLN Tfh and kidney graft-infiltrating CD4 T cells suggested a migration of dLN Tfh into the graft, even though other CD4 T cell subsets, such as peripheral T helper (Tph) cells have also been shown to produce IL-21 in inflamed peripheral tissues (25). To test the relationship between Tfh and intragraft IL-21–producing CD4 T cells, we performed allogenic kidney transplantation into *Il21*^Cre^*Rosa26*^Lox-STOP-Lox-YFP^ recipients and 20 days later sorted Tcon (CD4^+^CXCR5^–^YFP^–^, “LNTcon”), IL-21 fate-mapped Tfh from dLN (CD4^+^CXCR5^+^YFP^+^, “LNTfh21”), and intragraft fate-mapped CD4^+^YFP^+^ (“Graft21”) cells, and performed scRNA-Seq with an immune repertoire add-on analysis (Figure 2A and Supplemental Figure 2A). Using uniform manifold approximation and projection (UMAP) on all cells, we identified 19 distinct clusters separated by UMAP_1 and UMAP_2 dimensions (Figure 2B). The LNTfh21 cells separated distinctly from LNTcon and Graft21 cells, suggesting distinct transcriptional programs (Figure 2C). Similar separation of cell types was observed for the 2 biological replicates included in the data set (Supplemental Figure 2B). The LNTfh21 population separated into 5 distinct clusters (2, 9, 10, 17, and 19) and the Graft21 cells were separated into 3 distinct clusters (4, 7, and 15) (Figure 2B).

We next assessed which IL-21–producing clusters produce the highest amounts of current IL-21. Within the LNTfh21 population, *Il21* was highest in clusters 2 and 9 and in cluster 7 of the Graft21 cells (Figure 2D). As predicted, most cells and clusters in LNTfh21 expressed *Cxcr5,* whereas a proportion of cells in clusters 4 and 7 of Graft21 expressed *Cxcr5*, indicating a Tfh-like population. Moreover, a proportion of cells in clusters 4 and 7 also expressed *Bcl6*; however, the pattern was not completely overlapping with *Cxcr5* expression. All LNTfh21 and Graft21 cells and clusters expressed high levels of *Icos* and *Maf,* consistent with a Tfh/Tph phenotype. Expression of the coinhibitory receptor *Pdcd1* (PD-1) was equally high in Graft21 and LNTfh21 populations, whereas *Ctla4* was more highly expressed in Graft21 compared with LNTfh21. These data suggest that PD-1 and CTLA-4, known regulators of Tfh biology, may also regulate Tfh responses locally within allografts.

**Figure 3:**
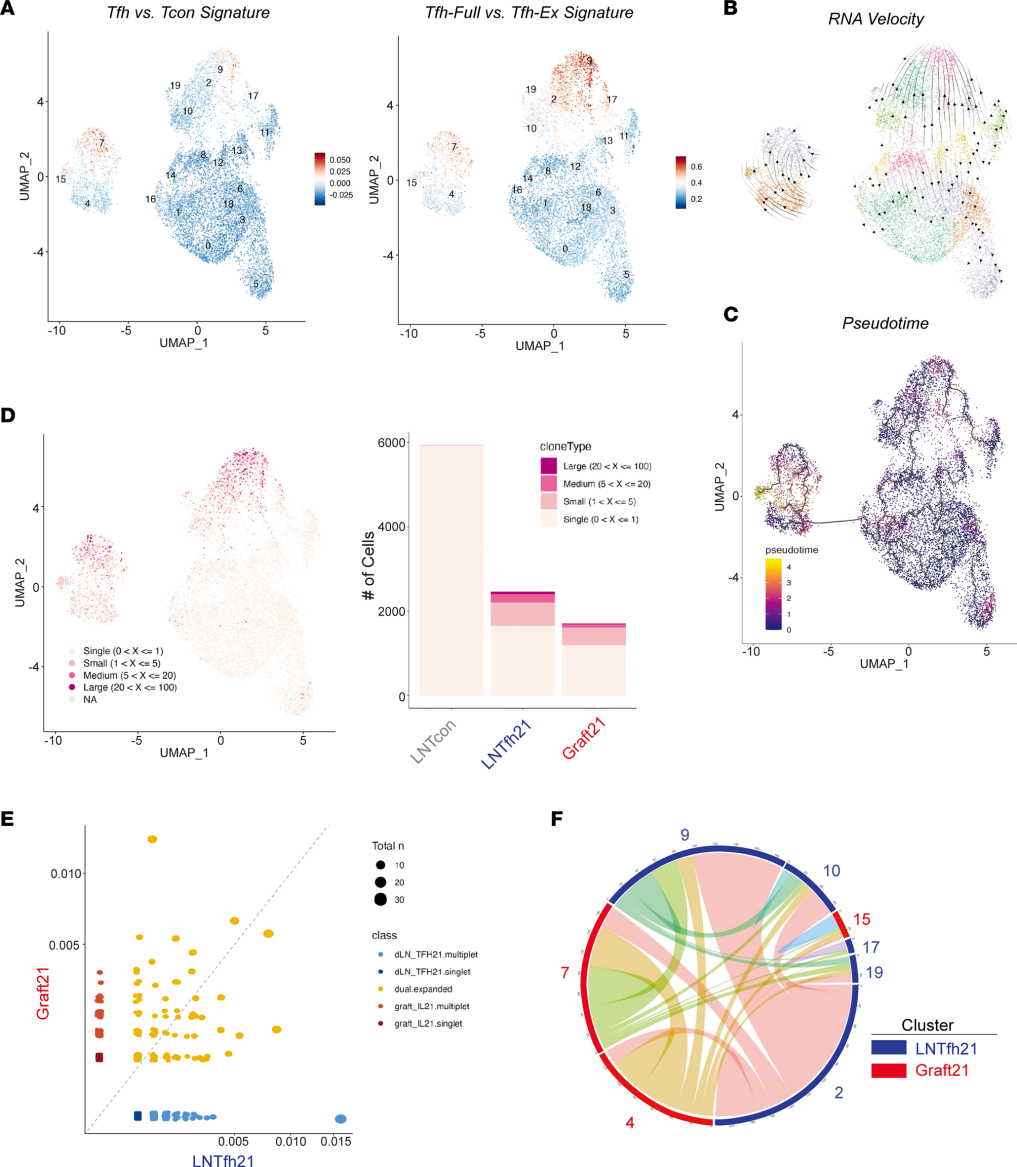
Overlapping origins and parallel developmental trajectories of LN and graft IL-21–producing Tfh cells. (**A**) UMAP with feature score annotation utilizing gene modules for “Tfh vs. Tcon signature” (left) and “Tfh-Full vs. Tfh-Ex signature” (right). (**B**) RNA velocity interrogation of cells in UMAP space. (**C**) Pseudotime trajectories of cells in UMAP space. (**D**) Clonal expansion indicated in UMAP space (left) and distribution of clonal expansion between groups (right). (**E**) Shared clones between LNTfh21 and Graft21 cells calculated by scRepertoire. (**F**) Circos plot showing shared clones among LNTfh21 and Graft21 clusters. Only expanded clones are included.

Differentially expressed gene (DEG) analysis showed cells in the LNTfh21 group expressed higher levels of Tfh-associated genes, including *Pdcd1, Maf, Cxcr3, Cxcr5*, and *Tox2,* while genes *Il17a, Junb, Ccl5, Ctla4, Ahnak,* and *Tnfrsf4* were expressed more highly in Graft21 cells (Figure 2E). The shared expression of individual Tfh genes between LNTfh21 and Graft21 prompted us to assess a broader gene module to identify Tfh programs in Graft21 cells. Using a “Tfh vs. Tcon” gene module, we found that clusters 7 and 15 of Graft21 cells had a transcriptional program consistent with Tfh cells, which even exceeded the values in most LNTfh21 cells themselves (Figure 3A). The heterogeneity of the Tfh module in LNTfh21 cells suggests multiple and complex cellular states in IL-21–producing Tfh cells 20 days after initial antigenic challenge. The expression of the Tfh gene module in clusters 7 and 15 and a sizable population of *Cxcr5/Bcl6*-expressing cells in clusters 4 and 7 together suggest that most Graft21 cells transcriptionally resemble Tfh cells. However, due to the transcriptional overlap of Tfh and Tph (which express many Tfh genes, except for Bcl6 and Cxcr5), it is possible that some Graft21 cells may also be Tph. Since the downregulation of IL-21 in Tfh may have functional consequences, we further validated a gene module (“Tfh-Full vs. Tfh-Ex”) which separates “effector” Tfh cells from Tfh cells that downregulate IL-21 and effector Tfh programs to become “Tfh-Ex” cells even though they maintain a surface phenotype consistent with a Tfh cell (24). Consistent with *Il21* transcript levels, low expression of the module was evident in clusters 4 and 15 of Graft21 and 10 and 19 of LNTfh21, indicating an “Ex-effector” Tfh population. RNA velocity analysis showed directional movement from IL-21 low clusters (4, 10) to IL-21–expressing clusters (7, 2, 9) for both Graft21 and LNTfh21 populations. This phenomenon was largely confirmed using pseudotime analysis, which also showed the most developed cluster to be cluster 15 of Graft21 cells (Figure 3, B and C).

To assess the origins of Graft21 cells, we interrogated the TCR sequences of cells in the scRNA-Seq data set. We found the most robust clonal expansion in clusters 2 and 9 of LNTfh21 as well as clusters 7 and 15 of Graft21 cells, all of which are clusters enriched in Tfh signatures (Figure 3D). We next assessed clonal overlap directly between groups and found a substantial amount of shared clones between LNTfh21 and Graft21 cells for both moderate and highly expanded clones (Figure 3E). When we evaluated shared expanded clones between clusters, we found all clusters had shared clones, including LNTfh21 and Graft21 clusters (Figure 3F). This phenomenon was also true between clusters no longer producing IL-21, such as cluster 10 of LNTfh21 and IL-21–producing clusters of LNTfh21 (cluster 2) and Graft21 (cluster 7). Moreover, there was substantial clonal overlap between cluster 7 (strong Tfh signature) and cluster 4 (weaker Tfh signature) of Graft21 cells, suggesting exchange between these clusters, consistent with RNA velocity data. Together, these data suggest parallel but identical developmental stage progression in Tfh cells in LNs and grafts, whereby IL-21–producing Tfh cells are seeded in both LNs and allografts from the same clones, and can regain expression of this cytokine and associative effector programs, possibly through activation signals.

### Costimulatory and anti-B cell therapy attenuates IL-21–producing Tfh cell development in LNs and allografts.

**Figure 4:**
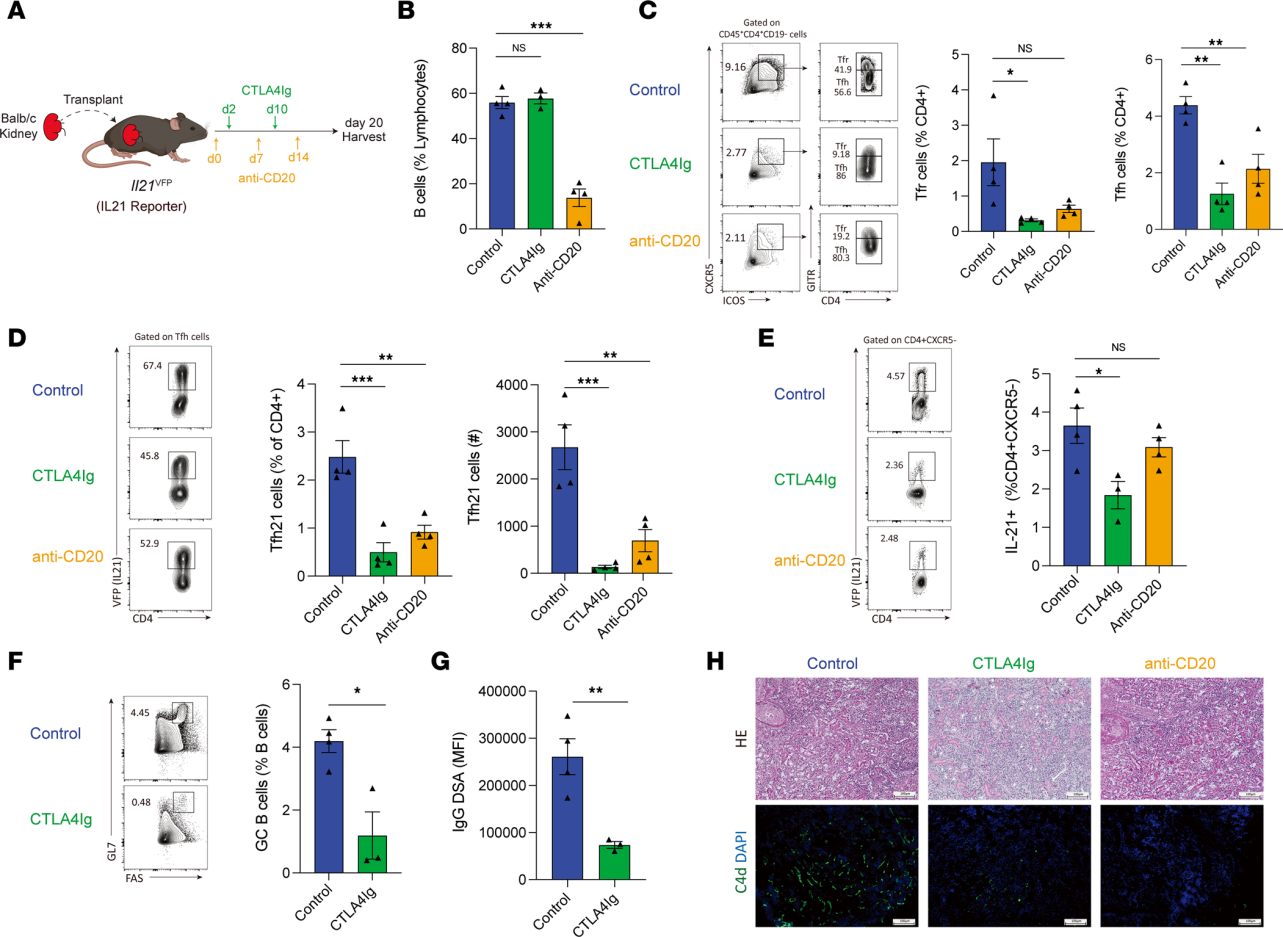
Transplant immunosuppression attenuates IL-21–producing Tfh cell development in LNs and grafts. (**A**) Schematic of CTLA-4Ig or anti-CD20 administration after allogeneic kidney transplantation. (**B**) Frequency of B cells in dLNs of recipient mice. (**C**) Gating strategy (left) and quantification (right) of Tfr (gated as CD4^+^CD19^–^CXCR5^+^ICOS^+^GITR^+^) and Tfh (gated as CD4^+^CD19^–^CXCR5^+^ICOS^+^GITR^–^) cells. (**D**) Quantification of IL-21–expressing Tfh cells in dLNs as a percentage of all CD4^+^ cells or as a total number. (**E**) Frequency (left) and total number (right) of IL-21–producing CD4^+^ T cells within kidney grafts. (**F**) Gating strategy (left) and frequency (right) of GC B cells from dLNs. (**G**) DSA IgG measurements from serum of transplanted mice. (**H**) Histological images of transplanted kidneys including H&E staining (top) and IF staining for C4d (bottom). Scale bars: 100 μm; original magnification: ×100. Data are combined from 2 independent experiments, *n* = 3–4 mice replicates per group. Student’s 2-tailed unpaired *t* test for 2-group comparisons for **F** and **G**, 1-way ANOVA with Tukey’s for multiple comparison for **B**–**E**. *P < 0.05; **P < 0.01; ***P < 0.001.

Diminishing CD28 signals endogenously through CTLA-4 expression in Tfh cells alters total Tfh development (26). Likewise, clinically targeted immunosuppression strategies, such as CTLA-4Ig and B cell depletion with anti-CD20 Abs, have been shown to alter Tfh populations in multiple diseases including transplantation (27–30). However, it is unknown whether such targeted immune modulatory strategies using costimulatory blockade or anti-B cell therapy alter the development of IL-21–producing Tfh cell subsets in the dLN and kidney grafts. Therefore, we performed experiments in which an allogeneic kidney was transplanted into *Il21*^VFP^ mice followed by immunomodulatory agents so that we could assess total and IL-21–producing Tfh populations (Figure 4A). As expected, only the anti-CD20 treatment resulted in substantially diminished frequencies of total B cells (Figure 4B). Both the CTLA4Ig and anti-CD20 treatments resulted in substantial reductions in the percentage of total Tfh cells in dLNs (Figure 4C) and in the percentage and total number of IL-21–producing Tfh cells (Figure 4D). In addition, both CTLA-4Ig and anti-CD20 treatments resulted in a reduction of IL-21–producing CD4^+^ T cells in LN (Supplemental Figure 3) and in the total number, but not frequency, of these cells in grafts, although only anti-CD20 reached statistical significance (Figure 4E). Since CTLA-4Ig attenuated Tfh cells, we next determined B cell alloimmunity in these settings. We found that CTLA-4Ig treatment substantially diminished the frequency of CD19^+^FAS^+^GL7^+^ GC B cells as well as serological IgG DSA responses (Figure 4, F and G). Mice receiving either treatment showed less substantial ABMR-characterized pathological damage, including tubulitis, perirenal capillaritis, and C4d deposition (Figure 4H). Together, these studies demonstrate that clinically used immunosuppression strategies attenuate IL-21–producing Tfh cell subset development and attenuate B cell alloimmunity after kidney transplantation.

### IL-21–producing Tfh cells are required for Ab-mediated rejection after kidney transplantation.

**Figure 5:**
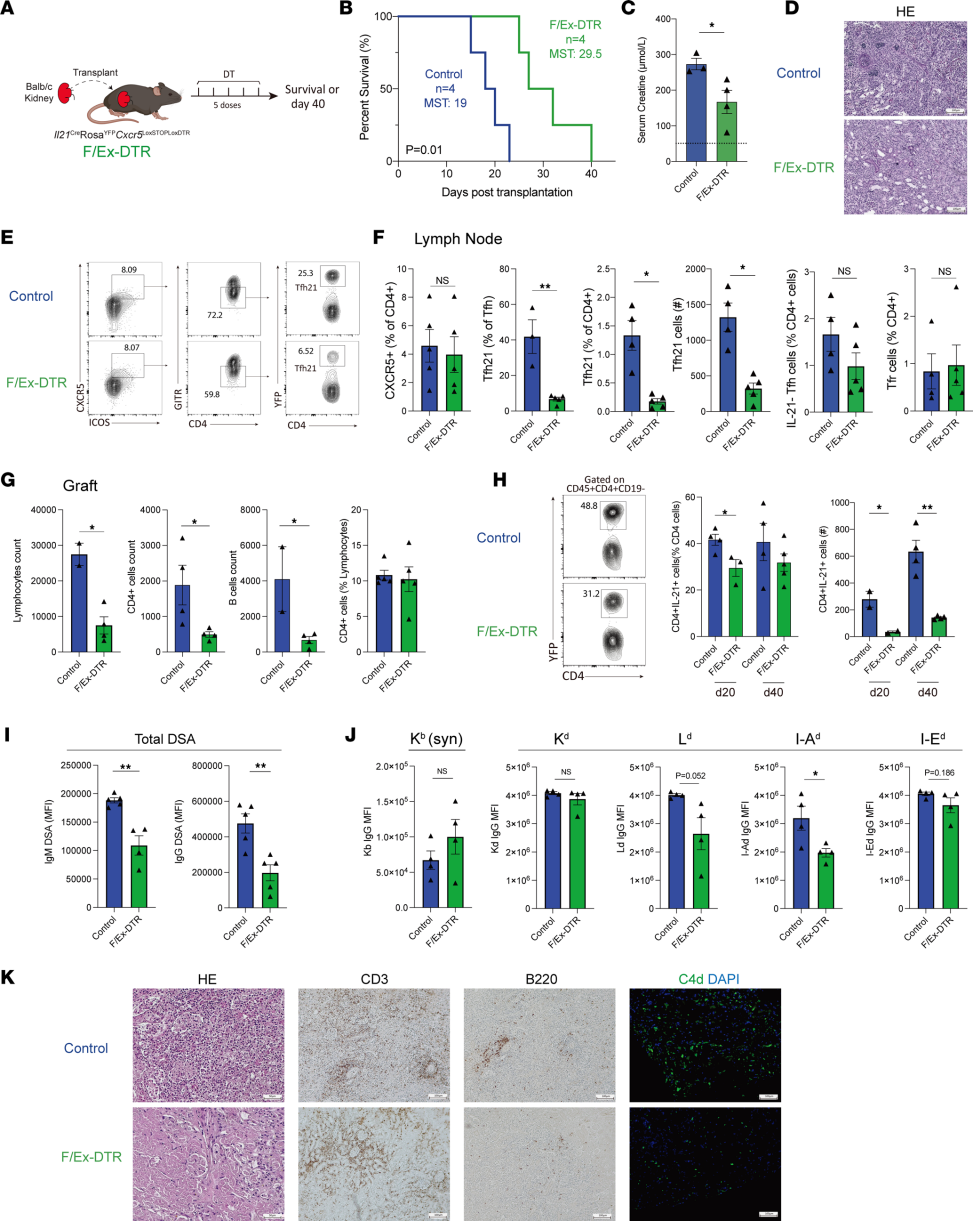
IL-21–producing Tfh cells are required for Ab-mediated rejection after kidney transplantation. (**A**) Schematic of IL-21–producing Tfh cell deletion using F/Ex-DTR mice. Control (*Il21*^Cre^*Rosa26*^Lox-STOP-Lox-YFP^*Cxcr5*^WT^) or F/Ex-DTR (*Il21*^Cre^*Rosa26*^Lox-STOP-Lox-YFP^*Cxcr5*^Lox-STOP-Lox-DTR^) mice were transplanted with a Balb/c kidney and cells deleted with the administration of DT. Life-sustaining, in **B**–**D**,or non–life-sustaining, in **E**–**K,** transplants were performed. (**B**) Survival of transplanted recipients between the control and the F/Ex-DTR group. MST, median survival time. (**C**) Serum creatinine levels of recipient mice. (**D**) Representative histological images of transplanted kidneys at the time of graft rejection. Scale bars: 100 μm; original magnification: ×100. (**E**) Gating strategy for IL-21–producing Tfh cells (gated as CD4^+^CD19^–^CXCR5^+^ICOS^+^GITR^–^YFP^+^) in the dLNs. (**F**) Quantification of CXCR5^+^ cells in total CD4^+^ T cells, IL-21–producing Tfh (Tfh21) and cell number, IL-21–nonproducing (IL-21^–^) Tfh cells, and Tfr (CD4^+^CD19^–^CXCR5^+^ICOS^+^GITR^+^) cells in dLNs. (**G**) Quantification of infiltrating lymphocytes, CD4^+^ T cells, and CD19^+^ B cells, as well as the frequency of CD4^+^ T cells, in transplanted grafts. (**H**) Quantification of IL-21–producing CD4^+^ T cells in kidney grafts at day 20 or 40 after transplantation. Left: frequency; and right: cell number. (**I**) Total IgG DSAs from serum of mice 40 days after transplantation. (**J**) Single antigen assays to assess IgG allospecificity. Syn, syngeneic. (**K**) Representative histological images of transplanted kidneys at postoperative day 40, including H&E, IHC staining for CD3 and B220, and IF staining for C4d. Scale bars: 100 μm; original magnification: ×100. Data are combined from 2 independent experiments with *n* = 3–5 mice per group. Kaplan-Meier survival analysis and a log-rank test for survival analysis for **B** and Student’s 2-tailed unpaired *t* test for 2-group comparisons for **C** and **F**–**J**. *P < 0.05; **P < 0.01.

To understand the precise contributions of dLN and graft IL-21–producing Tfh cells on rejection, we next used a system to specifically delete these cells *in vivo* after kidney transplantation. We used a mouse strain, which contains *Il21*^Cre^*Rosa26*^Lox-STOP-Lox-YFP^*Cxcr5*^Lox-STOP-Lox-DTR^ alleles so that any CXCR5^+^ Tfh cell that has *ever* expressed IL-21 is susceptible to deletion with the administration of diphtheria toxin (DT) (24). We refer to this strain as “F/Ex-DTR” for short since it deletes Fully differentiated *and* Ex-IL-21–expressing cells. We transplanted Balb/c kidneys into F/Ex-DTR (*Il21*^Cre^*Rosa26*^Lox-STOP-Lox-YFP^*Cxcr5*^Lox-STOP-Lox-DTR^) or control (*Il21*^Cre^*Rosa26*^Lox-STOP-Lox-YFP^*Cxcr5*^WT^) mice and gave 5 doses of DT post-allogeneic kidney transplantation to delete IL-21–producing Tfh cells (Figure 5A). In life-sustaining transplant surgeries in which native kidneys were rendered nonfunctional through a ureter obstruction method (12), deletion of IL-21–producing Tfh cells prolonged graft survival with a median survival of 29.5 days, compared with a median survival of 19 days in the control group (Figure 5B). The serum creatine levels were substantially decreased in the deleted mice, suggesting an improved graft function (Figure 5C). Although both groups of recipient grafts showed substantial monocyte infiltration, compared with the control mice, the F/Ex-DTR recipients had less tubulitis and peritubular capillaritis, which are typical pathological features of Ab-mediated rejection (Figure 5D). We also performed non–life-sustaining surgeries in which the mice were harvested on day 40 to allow an optimal amount of B cell alloimmunity and pathological rejection to occur (Supplemental Figure 4). We found that IL-21 fate-mapped Tfh cells, gated as CD4^+^ICOS^+^CXCR5^+^GITR^-^YFP^+^, were substantially deleted in F/Ex-DTR recipients, both as a frequency of Tfh cells as well as a frequency of CD4^+^ T cells (Figure 5, E and F and Supplemental Figure 5). We also found small decreases in IL-21 fate-mapped Tcon cells (CD4^+^CXCR5^–^YFP^+^) in F/Ex-DTR mice, likely reflecting a proportion of IL-21–producing Tfh cells that downregulate CXCR5 expression (Supplemental Figure 5A). Tfr cells, gated as CD4^+^CXCR5^+^GITR^+^ cells, were not changed between F/Ex-DTR and control mice (Figure 5F).

Within the allografts, the F/Ex-DTR mice showed less immune cell infiltration including reduced CD4^+^ T cells and CD19^+^ B cells (Figure 5G). The frequency, and more so total number of IL-21–producing CD4^+^ T cells in grafts, was lower in F/Ex-DTR mice at day 20 after transplantation, confirming that a proportion of the graft-infiltrating cells are Tfh-derived cells (Figure 5H). Although we did not observe differences in the frequency of these cells 40 days after transplantation due to biological variability, total numbers were substantially reduced. Next, we assessed serological DSA responses. We found that IgM and IgG DSA were reduced by approximately 60% in F/Ex-DTR compared with control mice at day 40 after transplantation (Figure 5I). We further used single alloantigen assays to assess individual alloepitopes. We found decreases in IgG Abs specific for both Class I and II antigens in the F/Ex-DT mice, although only anti–I-A^d^ IgG were statistically significant (Figure 5J). We also observed less graft damage associated with rejection including reduced mononuclear cell infiltration, tubulitis, peritubular capillaritis, and C4d deposition in the grafts of F/Ex-DTR mice compared with the control recipients at day 40 after transplantation (Figure 5K). Together, these data demonstrate that IL-21–producing Tfh cells substantially contribute to pathological rejection through controlling DSAs and ABMR.

### IL-21–producing Tfh cells promote LN and graft allogeneic GC B cell dynamics.

**Figure 6:**
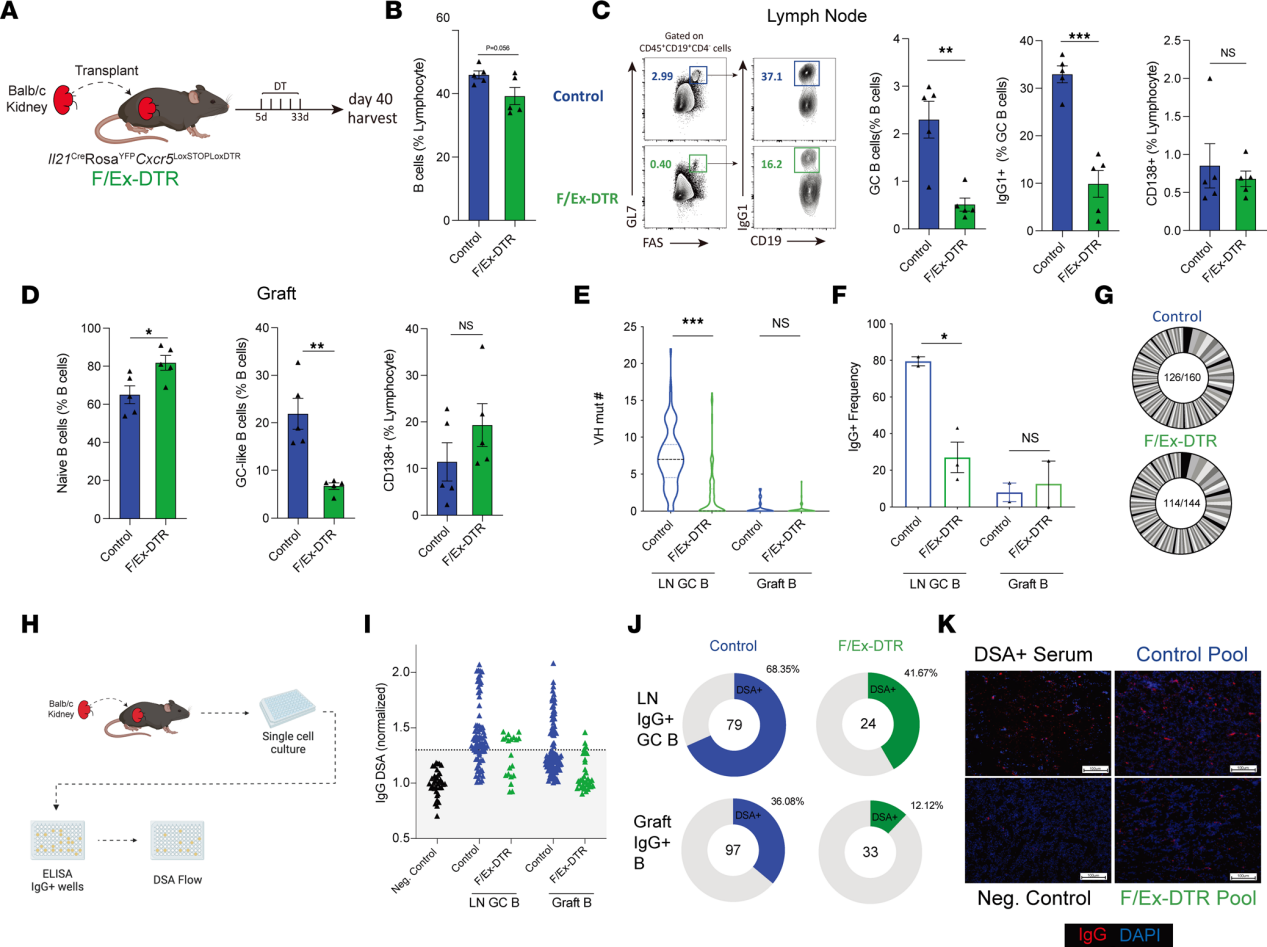
IL-21–producing Tfh cells mediate GC B cell dynamics in LNs and kidney grafts. (**A**) Schematic of IL-21–producing Tfh cell deletion using F/Ex-DTR mice. Control (*Il21*^Cre^*Rosa26*^Lox-STOP-Lox-YFP^*Cxcr5*^WT^) or F/Ex-DTR (*Il21*^Cre^*Rosa26*^Lox-STOP-Lox-YFP^*Cxcr5*^Lox-STOP-Lox-DTR^) mice were transplanted with a Balb/c kidney and cells deleted with administration of DT. (**B**) Quantification of total B cells (gated as CD45^+^CD19^+^CD4^–^) in dLNs. (**C**) Gating strategy (left) and quantification (right) of GC B cells, IgG1^+^ GC B cells, and CD138^+^ plasmablast/plasma cells in the dLN. (**D**) Quantification of naive B, GC-like B cells (CD45^+^CD19^+^CD4^–^GL7^+^FAS^+^), and CD138^+^ cells in kidney grafts. (**E**) Assessment of VH segment somatic hypermutation in LN GC B (CD19^+^GL7^+^FAS^+^) and Graft B (CD45^+^CD19^+^) cells. (**F**) Frequency of IgG^+^ cells from BCR sequencing in (**E**). (**G**) Clonal expansion of B cells in dLN. Same clone was defined as greater than 80% CDRH-3 aa sequence identity, same V-J segments, and CDRH-3 length. Numbers indicate total unique clones/total sequences. (**H**) Schematic of single B cell clonal assays. Single GC B cells from dLN or B cells from grafts were sorted and cultured with NB21 feeder cells for 6 days. Culture supernatants were prescreened for IgG positivity and further assessed for DSA reactivity. (**I**) DSA reactivity of individual IgG^+^ clones from LN GC B and graft B cells from either control or F/Ex-DTR mice 40 days after transplantation. Dotted line indicates level of detection threshold. (**J**) Frequency of DSA reactive clones (out of IgG^+^ clones). Numbers indicate the total number of IgG clones analyzed. (**K**) IF staining of allokidneys using top 5 alloreactive dLN clones from **J**. Positive signal: red; DAPI: blue. Scale bars: 100 μm; original magnification: ×100. Data are combined from 2 independent experiments with *n* = 4–5 mice per group. Student’s 2-tailed unpaired *t* test for **B**–**D** and Mann-Whitney test for **E** for 2-group comparisons. *P < 0.05; **P < 0.01; ***P < 0.001.

Since we found that deletion of IL-21–producing Tfh cells resulted in reduced pathological rejection and serological DSA responses, we next wanted to determine how these cells differentially contribute to B cell alloimmunity, both systemically and locally, within grafts. To do this we performed allogeneic kidney transplantation into F/Ex-DTR or control mice and administered DT. We harvested dLN and kidney grafts 40 days after transplantation and assessed effector B cell responses (Figure 6A). Deletion of IL-21 fate-mapped Tfh cells resulted in robust attenuation of GC B cells as well as the proportion of IgG1^+^ B cells within the GC B population (Figure 6, B and C). In contrast, CD138^+^ plasmablasts/plasma cells were not affected by deletion of IL-21–producing Tfh cells. Although the reason for discordant changes between GC and plasma cell compartments is unclear, it is possible that many plasma cells may be preexisting, or may not be generated from a GC process. Within the graft, the proportion of GL7^+^ GC-like B cells (activated B cells) was also substantially attenuated in deleter mice without substantial changes in CD138^+^ plasmablast/plasma cells (Figure 6D).

To understand the specific roles of IL-21–producing Tfh cells in promoting SHM of alloimmune B cells in more detail, we sorted GC B cells from the dLN and total B cells from kidney grafts and performed B cell receptor (BCR) sequencing. GC B cells from the dLN of transplanted control mice had a substantial amount of SHM on day 40 after transplantation, consistent with recent reports (22) (Figure 6E). Deletion of IL-21–producing Tfh cells resulted in a substantial reduction in the amount of SHM in LN GC B, but not graft-infiltrating B cells, which were largely germline for both control and deleter mice. In addition, the proportion of IgG-switched cells within the LN GC B cell population was reduced substantially in deleter mice, suggesting the IL-21–producing population of Tfh cells is also essential for CSR in GCs (Figure 6F). In control mice with no Tfh deletion, both IgM and IgG clones showed evidence of SHM (data not shown). We also assessed clonal diversity by identifying unique clones based on greater than 80% CDRH-3 (the third complementarity determining region of Ab heavy chains) aa sequence similarity, identical CDRH-3 segment usage, and identical length. We found a moderate amount of clonal expansion in the control LN GC B cell compartment, but this was not substantially altered in deleter mice (Figure 6G). To assess GC B cell dynamics in more detail, we utilized a single B cell culture system in which an individual B cell clone is sorted and cultured with feeder cells that allow B cell expansion and Ab secretion without inducing SHM or switching (Figure 6H). We then screened clones for IgG positivity and further screened IgG^+^ clones for DSA specificity. In control mice, approximately 68% of LN IgG^+^ GC B cells showed DSA reactivity, whereas only approximately 36% of graft IgG^+^ B cells were allospecific (Figure 6, I and J). In F/Ex-DTR mice, there was an approximately 30% reduction in the frequency of alloreactive clones in the IgG^+^ LN GC compartment and an approximately 50% reduction in the IgG^+^ graft B cell compartment (Figure 6J). These data suggest that IL-21–producing Tfh cells in LN and allografts promote DSA-specific–switched B cells. To confirm that the DSAs produced by these clones were in fact capable of binding to alloantigens to mediate ABMR, we pooled the top 5 IgG^+^ DSA clones from the LN and graft and used them to stain allogeneic kidney sections. We found that the DSA from the control group was capable of binding allogeneic kidney sections similarly to deleter mice (Figure 6K). Together these data indicate that IL-21–producing Tfh subsets are essential for ABMR after kidney transplantation by promoting DSA-specific GC responses both in dLNs and locally within allografts.

## Discussion

A fundamental strategy to treat ABMR after kidney transplantation is to drastically reduce DSA; however, mechanisms controlling DSA are relatively unknown. Here, we uncovered that a specific subset of Tfh cell, marked by IL-21 production, has essential roles in controlling DSA and ABMR. These IL-21–producing Tfh cells undergo parallel development in both dLNs as well as in kidney allografts and share common progenitors. Clinically used immunosuppression strategies, such as CTLA4-Ig and B cell depletion, alter the development of these cells. Functionally, IL-21–producing Tfh cells control alloantibodies by promoting GC formation, SHM, and Ab CSR. Although Tfh cells promote pathogenic DSA responses in both dLN and in allografts, GC B cell dynamics are distinct in each of these locations with graft-infiltrating allogeneic B cells having less affinity maturation. Therefore individual subsets of Tfh cells can have multifaceted roles in inducing ABMR after kidney transplantation.

In renal transplant patients with chronic rejection, IL-21–capable Tfh populations (Tfh2 and Tfh17) in the circulation were found to correlate with the occurrence of ABMR (31). Donor-specific IL-21–producing total T cells were also found to be enhanced in patients who developed early and late rejection (32). By using the IL-21 fate-mapper and reporter mice, we found that only approximately 60% of Tfh cells have ever produced IL-21 in dLNs during ABMR after kidney transplantation. Surprisingly, we found IL-21–producing Tfh-like cells also within allografts. By scRNA-Seq, we found the IL-21–producing Tfh cells in dLN are marked by Tfh-effector genes, including *Cxcr5*, *Pdcd1*, *Maf*, *Cxcr3*, and *Icos*, and shared similar transcriptional features with graft-infiltrating IL-21^+^ Tfh-like cells. The expression of *Ahnak* in graft Tfh-like cells was of interest because it is also expressed in kidney graft-specific B cells in mice and humans (22, 33). Interestingly, in both LNs and allograft IL-21–producing Tfh cells, we observed distinct IL-21 active and nonactive clusters, suggesting Tfh cells exist in distinct functional states. Importantly, transcriptional programming between these clusters suggests similar mechanisms for initiating functionality in Tfh cells in both LNs and allografts. However, some transcriptional programming was tissue-specific for the allograft, including *Il17a* and *Junb* expression. JunB was reported to contribute to Th17 cell pathogenicity (34), and Th17 cells are abundant in allografts during acute rejection and are associated with graft failure (35).

CTLA-4–mediated inhibition of CD28 signals can control B cell responses by modulating Tfh cells (26–28). We found that clinically relevant immunosuppression with CTLA-4Ig attenuates IL-21–producing Tfh cells. Similar results were found with B cell depletion using anti-CD20 mAb. In patients, circulating activated Tfh cells were found to be reduced in patients treated with CTLA-4Ig with a lower proportion of DSAs (29). Furthermore, selective deletion of IL-21–producing Tfh cells prolonged graft survival and improved graft function, suggesting a potential clinical relevance and therapeutic strategy for improving kidney graft survival. We showed that these cells have multifaced roles in promoting allogeneic B cell responses after kidney transplantation. Within the dLN, IL-21–producing Tfh cells control allogeneic GC formation, CSR, and SHM. Both donor-specific IgG and IgM Abs were attenuated in deleter mice, suggesting a reduced activation or response of naive B cells to the allogantigen, which initially produces IgM before class switching. Considering the context of transplantation and the alloimmune response, the reduction in IgM levels might also imply a diminished or delayed primary immune response to the graft. Although IgM Abs are generally considered less pathogenic compared with IgG, they are crucial for the early immune response. The IL-21–producing Tfh cells appear to have a broader role in GC dynamics, affecting not only the class-switched IgG response but also the generation of IgM Abs by influencing early alloimmune responses and B cell maturation within the GC, shaping the overall donor-specific Ab response. Interestingly, IL-21–producing Tfh cells also promote allogeneic IgG^+^ B cells acquiring a GC phenotype within allografts but are not able to induce SHM. The difference in functionality may be due to alterations in the Tfh transcriptional program dictated by the allograft microenvironment and/or intrinsic alterations in allograft B cells. Further experiments will need to be performed to explore these areas in more detail. Our research suggests a potential avenue for future investigation, based on the distinct roles Tfh cells can play in different immune contexts. Here, we found IL-21–producing Tfh cells have roles in 2 separate effector sites of allo-responses. During some viral infections, IL-21 has been reported to be less essential for acute humoral immunity due to compensation from other cytokines (36). Given the complex and diverse nature of Tfh cells, it is plausible that manipulating a specific subset, like IL-21–producing Tfh cells, would be more susceptible to effects on alloimmunity than antiviral immunity. The ability to selectively modulate these responses could have important implications for transplant recipients who need to maintain effective anti-viral immunity while suppressing harmful alloimmune responses. Therefore, targeting IL-21–producing Tfh cells may potentially treat AbMR while uncoupling allo- and antiviral immunity. Our data suggest that altering IL-21–producing Tfh cells may have a synergistic effect in attenuating allogeneic B cell responses both in dLNs and within kidney allografts and may avoid the undesired effects of broad immunosuppression.

## Methods

### Mice.

Balb/c mice were purchased from Jackson Laboratories. *Il21*^Cre^ was a gift from Uta Hoepken (Max Delbrück Center for Molecular Medicine, Berlin, Germany) and has been published previously (37), *Rosa26*^Lox-STOP-Lox-YFP^ and *Il21*^VFP^ were from Jackson Laboratories, and *Cxcr5*^Lox-STOP-Lox-DTR^ mice on the C57BL/6 background were published previously (17).

### Kidney transplantation.

The allogeneic kidney transplantation procedure was performed as described previously (2, 12, 22). Briefly, the left donor kidney was transplanted with a ureter into the abdominal cavity of the recipient mice. The artery and vein of the donor’s kidney were anastomosed to the recipient’s abdominal aorta and vena cava in an “end-to-side” manner using 10-0 sutures. The graft ureter was embedded into the recipient’s bladder to reconstruct the urinary tract. Typical surgeries lasted 2 hours, with an anastomotic period of 30 minutes. The recipients were harvested on day 20 or 40 after transplantation as indicated. For life-sustaining surgery, the ureter of recipient native kidneys was ligated on postoperative days 2 to 4. The grafts and serum were harvested at the time of graft failure with signs, including lethargy, decreased mobility, and ruffled hair. The mouse creatinine assay kit (Crystal Chem) was used to assess the graft function.

### Treatments.

On days 2 and 10 after transplantation, 250 μg CTLA4-Ig (Abatacept, Bristol-Myers Squibb) was given by i.p. injection. For the B cell deletion, 250 μg of anti-mouse CD20 (BioLegend, clone SA271G2) was given by i.p. injection on days 0, 7, and 14 after transplantation. For deletion experiments, mice were i.p. administered 0.5 μg of DT in PBS at indicated time points.

### Pathology and immunofluorescence.

Kidney grafts were harvested at designated time points and either immediately submerged in 10% formalin for paraffin embedding or preserved in OCT; and 4 μm sections were used for HE staining. IHC and immunofluorescence (IF) staining were performed as published previously (38). The Abs used included anti-CD3(Cell Signaling Technology, D4V8L), B220 (BioLegend, RA3-6B2), and C4d (Novus Biologicals, 16D2). To test the ability of DSA produced from single-cell cultures to bind to alloantigens, supernatants from the highest 5 DSA-positive clones were pooled and used to bind allogeneic native Balb/c kidney sections for 24 hours at 4°C. Then, primary Ab anti-IgG (SouthernBiotech) was used to detect the IgG. Images were captured on a ZEISS Axiolab 5 microscope.

### Flow cytometry.

Spleen, dLNs and grafts were prepared as single-cell suspensions with grafts undergoing collagenase digestion using collagenase I (Worthington). Graft-infiltrating lymphocytes were separated using ficoll-paque (Cytiva) according to the manufacturer’s instruction. Abs were all from BioLegend except where noted, including anti-CD4 (RM4-5), anti-CD19 (6D5), anti-ICOS (15F9), anti-CXCR5 (L138D7), anti-PD-1 (RMP1-30), anti-GITR (DTA-1), anti-CD45 (30-F11), anti-CD38 (90/CD38), anti-GL7 (BD Biosciences, GL-7), anti-FAS (BD, JO2), anti-IgG1 (BD Biosciences, A85-1), and anti-IgG2 (RMG2a-62 and RMG2b-1). Surface marker staining was performed at 4°C for 30 minutes. Some samples were intracellularly stained using the Foxp3 Fix/Perm buffer set according to the manufacturer’s instructions (eBioscience). Flow cytometry acquisition was performed on a Cytek Aurora spectral analyzer and analyzed with FlowJo v10.

### DSA measurements.

After 5 × 10^5^ splenocytes from WT Balb/c or C57Bl/6 mice were incubated with serum samples (1:50 dilution for IgG, 1:25 dilution for IgM) or supernatant from single-cell cultures (not diluted) for 30 minutes, they were then washed and stained with anti-CD19 (BioLegend, 6D5) in the presence of Fc-block (BioLegend) and subsequently stained with FITC-labeled anti-IgG or anti-IgM. For single alloantigen assays, MHC beads (K^b^, K^d^, L^d^, I-A^d^, and I-E^d^) and I-E^d^ were incubated with serum (1:50 dilution) at 4°C for 1 hour. FITC-labeled anti-IgG was used to detect bound Abs. Samples were analyzed on a Cytek Aurora flow cytometer with spleen cells from C57BL/6 syngeneic mice as a staining control. DSA was quantified by measuring the IgG or IgM signal on CD19^+^ splenocytes, which is expressed as MFI.

### Single B cell cultures.

Single GC B cell cultures were performed as previously described (39). Single GC B cells (sorted as CD45^+^B220^+^CD4^–^GL^–^7^+^CD38^lo/–^) from LNs and total B cells (CD45^+^B220^+^CD4^–^) from kidney grafts were sorted into 96-well plates and cultured with 1 x 10^3^ NB21 feeder cells (a gift from Garnett Kelsoe, Duke University, Durham, North Carolina, USA) for 6 days. IgG-positive clones were identified by ELISA and then further assessed for DSA reactivity, as above.

### BCR sequencing.

*Igh* sequencing was performed as previously described (40). In brief, single LN GC B cells (gated as CD45^+^B220^+^CD4^–^GL^–^7^+^CD38^lo/–^) and intragraft B cells (gated as CD45^+^B220^+^CD4^–^) were sorted into 96-well PCR plates containing 5 μL TCL lysis buffer with 1% 2-mercaptoethanol. After extraction, RNA was reverse transcribed into cDNA using an oligo (dT) primer. *Igh* transcripts were amplified and PCR products were barcoded and sequenced. The alignment of V(D)J segments to *Igh* sequences was performed based on the international ImMunoGeneTics information system (IMGT). The IMGT and Vbase2 databases were utilized to detect the VH mutations. Clones were defined as functional rearrangements that shared the same VH and JH segments, the same CDR-H3 length, and at least 80% similarity in CDR-H3 aa sequences.

### scRNA-Seq and analysis.

*Il21*^Cre^*Rosa26*^Lox-STOP-Lox-YFP^ mice on the C57BL/6J background were transplanted with Balb/c kidneys, which were harvested 20 days later. Single-cell suspensions from the graft and dLN were stained with flow cytometry Abs and distinct barcoded Abs (Cell-Hashing Ab, TotalSeq-C, BioLegend) as previously described (41). Tfh21 (CD4^+^CXCR5^+^YFP^+^) cells from dLNs and graft-infiltrating CD4^+^YFP^+^ cells were sorted along with LN Tcon cells (CD4^+^CXCR5^–^YFP^–^). Two cohorts (*n* = 2 per cohort) were performed for each of the 3 cell types. Cells from each condition were pooled together and resuspended in PBS 0.4% BSA at a concentration of 2,000 cells/μL. Samples were subsequently loaded onto a single lane (Chromium chip K, 10X Genomics) followed by encapsulation in lipid droplets (Single Cell 5′ kit V2, 10X Genomics) at the Brigham and Women’s Hospital Single Cell Genomics Core. cDNA and library generation were performed according to the manufacturer’s protocol. The 5′ mRNA library was sequenced to an average of 50,000 reads per cell, whereas the V(D)J library and hashtag oligo (HTO) (Cell Hashing Abs) library were both sequenced to an average of 5,000 reads per cell, all using Illumina Novaseq. Reads were processed with Cell Ranger, and quantification was performed using the STAR aligner against the Mm10 transcriptome. CellRanger output data were loaded into the R programming environment and analyzed with the Seurat package. Sample demultiplexing and doublet exclusion were performed with the HTODemux function, and only singlets were selected for further analysis. Additional quality-control filtering was performed, imposing as thresholds: unique molecular identifier (UMI) counts greater than 1,000 and less than 15,000, gene counts greater than 500, log-transformed genes per UMI greater than 0.8, and mitochondrial RNA content less than 10%. Count data were subjected to normalization and variance stabilization using the SCTransform function (version 2) based on the 3,000 most variable genes and by concomitantly regressing variability from mitochondrial and ribosomal mapping percentages. Additional filtering based on identity was applied after comparison of each cell with the Immunologic Genome Project data set (using the SingleR pipeline) to exclude contaminating B, CD8^+^ T, and NK cells (42). UMAP was used for dimensionality reduction according to the standard Seurat pipeline, but TCR-related genes were excluded from the list of variable features to avoid clustering based on clonotype. DEGs between conditions were computed with the DESeq2 pipeline after collapsing count data within each HTO (pseudobulk analysis); significance was inferred at a *P* value of 10^–5^ along with a log_2_ fold change (FC) threshold of ± 1. Module scores were calculated with the AddModuleScore function, using as input gene sets comprising upregulated genes in Tfh compared with Tconv cells derived from our bulk RNA-Seq data set, or a list of upregulated genes in TfhFull compared with TfhEx cells, from a previous single-cell experiment (24). TCR clonotype analysis was performed with the scRepertoire package (43). Raw data have been deposited in the NCBI’s Gene Expression Omnibus database (GEO) under accession number GSE241181.

### In vitro functional assays.

IL-21 fate-mapped, IL-21 non–fate-mapped Tfh cells, and B cells were isolated from spleens of IL-21 fate mapping mice transplanted with allogeneic kidneys for 20 days. In vitro assays were performed by coculturing 5 × 10^4^ B cells with 3 × 10^4^ Tfh and anti-CD3/IgM in 96-well U bottom plates. Cultures were maintained in complete medium for 4 days.

### Statistics.

The Student’s 2-tailed unpaired *t* test or Mann-Whitney test was performed to determine the significant difference between 2 groups using GraphPad Prism, version 9 (GraphPad Software). One-way ANOVA followed by Tukey’s test was used for multiple comparisons. Kaplan-Meier survival analysis and a log-rank test were performed to compare the graft survival. Significance was inferred at a *P* value of less than 0.05. The number of mice per group, the number of replicates per experiment, summary statistics, and measures of dispersion are indicated in the legend of each Figure. Values shown in the graphs represent the SEM.

### Study approval.

All animal research was performed according to Brigham and Women’s Hospital IACUC and NIH guidelines.

### Data availability.

All data are available in the main text or supplementary material. Values for all data points are provided as a Supporting Data Values file.

## Author contributions

HZ designed and performed experiments, acquired and analyzed data, and wrote the manuscript. CBC and MAP performed experiments and analyzed data. EDB, GR, PC, JML, and IS performed experiments. SGT, RA, BRB, and ASC provided key technical help. PTS designed experiments, analyzed data, and wrote the manuscript. All authors edited the manuscript.

## Supplementary Material

Supplemental data

Supporting data values

## Version 1 10/23/2023

Electronic publication
