# Supplementary material for: IL-21–producing effector Tfh cells promote B cell alloimmunity in lymph nodes and kidney allografts
Source: JCI Insight. 2023 Oct 23;8(20):e169793. doi: 10.1172/jci.insight.169793 (PMC10619486; doi:10.1172/jci.insight.169793)
Supplement: Supplemental data [file jciinsight-8-169793-s262.pdf]

Supplemental material

Figure S1

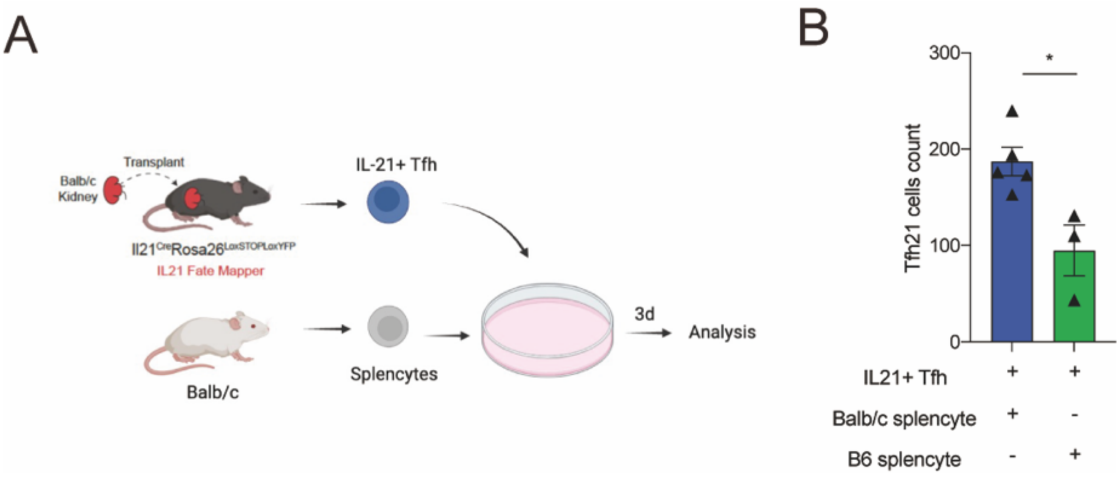

Figure S1. The donor-reactivity of IL-21 producing Tfh cells was detected by mixed lymphocyte reactions.

(A): Schematic of Tfh cell MLR assay. IL-21 producing Tfh cells (30k cells/well) sorted from allogeneic transplantation recipients were culture with syngeneic (B6 mice) or allogeneic (Balb/c mice) donor splenocytes (100k cells/well) for 3 days.

(B): Quantification of Tfh cells as a total number in the culture system.

Figure S2

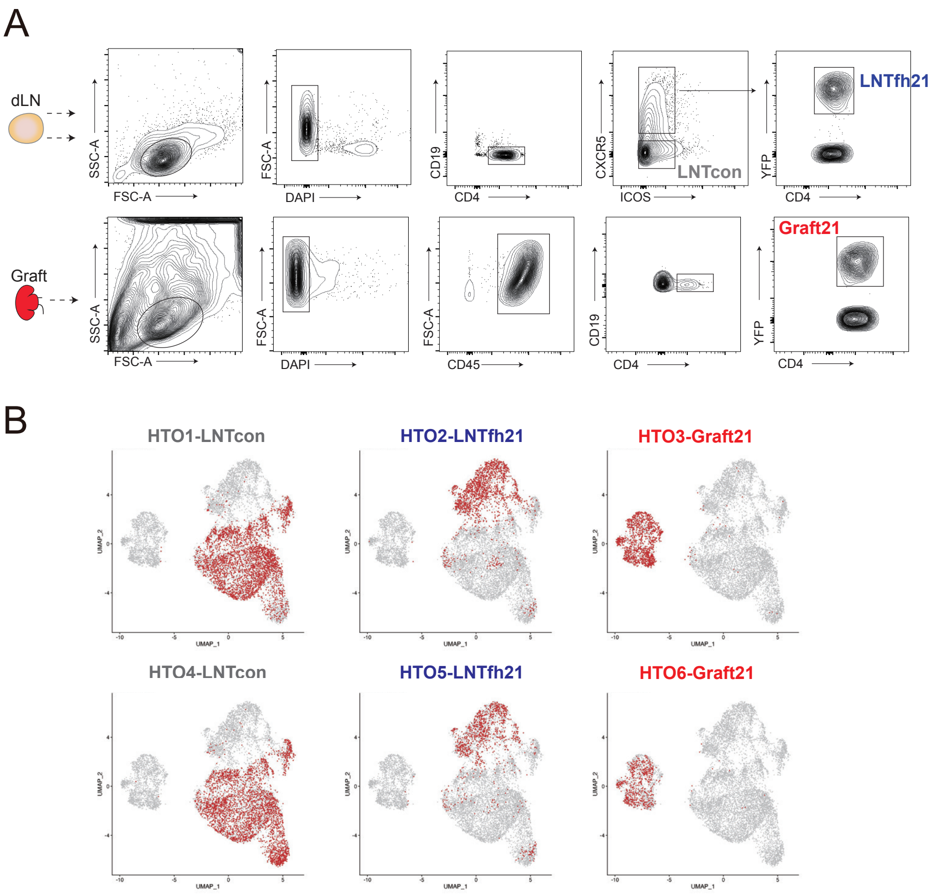

Figure S2. Sorting strategy and individual sample assignment in the single cell RNA-seq experiment.

(A) Sorting strategy for indicated populations of Tcon (CD4+CXCR5-YFP-, "LNTcon"), IL-21 producing Tfh (CD4+CXCR5+YFP+, "LNTfh21") from dLN and intragraft CD4+YFP+ ("Graft21") that underwent scRNAseq analysis.

(B) Assignment of cells to individual samples based on hashtag oligo (HTO) differential expression.

Figure S3

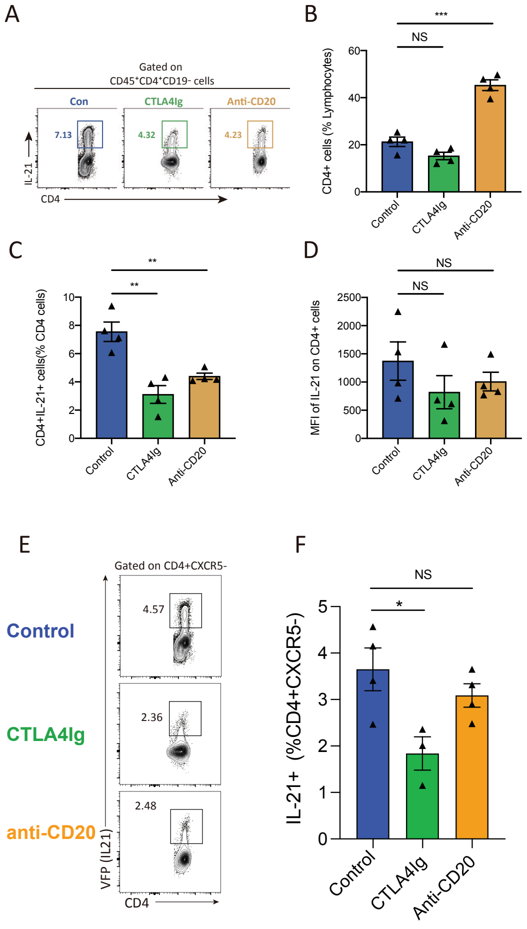

Figure S3. CTLA-4Ig and B cell deletion inhibited IL-21+ proportion of T cells and DSA production.

Gating strategy (A) and frequency of $CD4^+$ T (B), $CD4^+IL\text{-}21^+$ T cells (C) and IL-21 expression levels on total CD4+ T cells (D). Representative gating (E) and quantification (F) of the frequency of $IL\text{-}21^+$ cells in $CD4^+CXCR5^-$ T cells.

n=3-4 mice replicate per group. Statistics: Student's two-tailed unpaired T test, NS: not significant; **: P < 0.01; ***: P < 0.001.

Figure S4

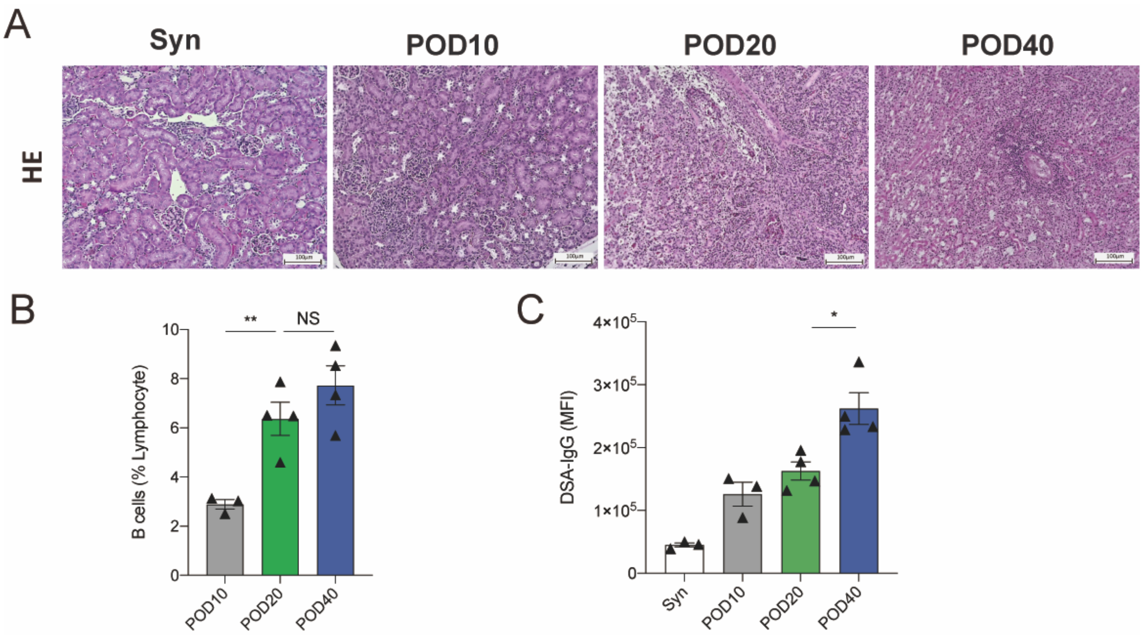

Figure S4. Kidney allograft rejection kinetics.

(A): Representative histological images of transplanted kidneys for syngeneic (Syn), allogeneic postoperative days 10, 20, and 40. The results showed a progressive increase in monocyte infiltration in the grafts over time. Notably, indications of tubulitis and glomerulonephritis emerge by day 10 and become severe by day 40 post-transplantation. HE: hematoxylin and eosin staining. Magnification: 100×, scale bars: 100µm.

(B): Quantification of graft infiltrating CD19+ B cells in total lymphocytes at different timepoint.

(C) Total IgG DSA from the serum of syngenetic and allogeneic recipients 10, 20, and 40 days after transplantation.

Figure S5

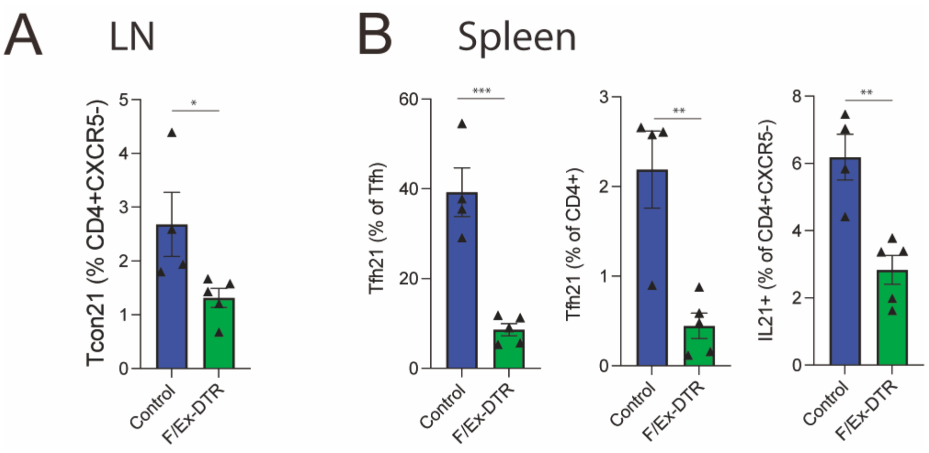

Figure S5. IL-21 producing Tfh cells were deleted in F/Ex-DTR mice.

(A): Quantification of Tcon21 ($CD4^{+}CXCR5^{-}YFP^{+}$) in CD4+CXCR5- cells in dLNs.

(B): Quantification of IL-21 producing Tfh (Tfh21) and Tcon21 ($CD4^{+}CXCR5^{-}YFP^{+}$) in spleens of recipient mice.
